# Intestinal parasitosis among HIV/AIDS patients who are on anti-retroviral therapy in Kombolcha, North Central, Ethiopia: a cross-sectional study

**DOI:** 10.1186/s13104-018-3726-6

**Published:** 2018-08-25

**Authors:** Daniel Gebretsadik, Haftay Haileslasie, Daniel Getacher Feleke

**Affiliations:** Department of Medical Laboratory Science, College of Medicine and Health Sciences, Wollo University, Dessie, Ethiopia

**Keywords:** Kombolcha, HIV/AIDS, ART, Intestinal parasites

## Abstract

**Objective:**

Human immunodeficiency virus (HIV) infected patients are highly vulnerable to microbial and parasitic diseases due to the immune-suppression. This study aimed to determine the prevalence and assess the associated risk factors of intestinal parasites in HIV/AIDS patients who are under anti-retroviral therapy in Kombolcha, North-Central Ethiopia.

**Result:**

A total of 223 HIV sero-positive individuals who are on ART in Kombolcha Health Centre were examined for intestinal parasites. Of the total study participants 153 (68.6%) were females, 205 (91.9%) were urban resident and 116 (52.0%) were married. Intestinal parasites were detected in 31 (13.9%) of the 223 study participants. Nine different intestinal parasite species were detected and the most prevalent intestinal parasite detected was *E. histolytica*, which accounts 7.2% (16/223). Majority of study participants had the habit of washing their hand before meal and after toilet 215 (96.4%) and most of the study participants 126 (56.5%) had private toilet.

**Electronic supplementary material:**

The online version of this article (10.1186/s13104-018-3726-6) contains supplementary material, which is available to authorized users.

## Introduction

Human immunodeficiency virus (HIV) poses a great international challenge. Globally, an estimated 33 million adults and children are living with the virus. Among the regions, Sub-Saharan Africa was the most affected. Human immunodeficiency virus (HIV) infected patients are highly vulnerable to microbial and parasitic diseases due to the immune-suppression [1].

Parasitic infections are widely spreaded human public health problem worldwide [2]. Intestinal parasites affected about two billion people, of whom 300 million suffer from associated severe morbidity [3]. Intestinal parasitic infections are associated with ignorance/illiteracy, contamination of food and drinking water and unhygienic lifestyle including indiscriminate disposal of sewage and other wastes [4].

Intestinal parasites are endemic in many regions of the world where HIV/AIDS is also prevalent (19, 102, 20, 39, and 41). In developing countries, acute gastroenteritis due to intestinal parasites cause illness and kills millions of AIDS patients annually [2].

Anti-retroviral treatment increases the length and quality of life; it also decreases the incidence of opportunistic infections in HIV/AIDS patients [5]. However, patients on ART with low CD4+ counts may suffer from intestinal parasitic infections (model). Previous studies reported that the proportion of opportunistic pathogens were higher in patients with CD4 count < 200 cells/μl as compared with patients with CD4 count > 200 cells/μl [6, 7].

Like many developing countries, intestinal parasites are widely distributed in Ethiopia [4]. Few studies investigated intestinal parasites prevalence and associated factors on HIV/AIDS patients in Ethiopia and as far as we know there was no study conducted in Kombolcha, North central Ethiopia. This study aimed to determine the prevalence and assess the associated risk factors of intestinal parasites in HIV/AIDS patients who are under anti-retroviral therapy in Kombolcha, North-Central Ethiopia.

## Main text

### Methods

#### Study area and period

This study was carried out at Kombolcha Health Centre, North-Central Ethiopia. Kombolcha is a town located at an altitude of about 1857 meters above sea level and 376 km North-East of Addis Ababa, the capital of Ethiopia. In 2015, the estimated total population of Kombolcha town was 126,144; of whom 65,918 were female. Majority of population lives in urban areas of the town. The mean annual rainfall is 841.1 mm; the maximum and minimum annual mean temperature is 27.4 °C and 12.9 °C, respectively. The Kombolcha Health Centre is the preferred health institution by the local population and as such has a very high patient load. Kombolcha Health Centre had 2725 HIV patients who are on ART until the end of 2016.

#### Study design

This institution based cross-sectional study was conducted from June 2016 to August, 2016 in Kombolcha Health Centre, North-Central Ethiopia.

#### Sampling technique and sample size determination

HIV/AIDS patients who fulfilled the inclusion and exclusion criteria were selected by systematic random sampling method using the ART clinic registration book as a sampling frame. The sample size was determined using the single proportion population formula. It was calculated by using a prevalence of 17.6% [8] with a margin of error of 0.05 and a confidence level of 95%. In line with this, 223 study participants were recruited.

#### Ethical consideration

The study was conducted after obtaining ethical clearance from Wollo University ethical committee. A written consent form was used to ask the willingness of the study participants. Participants with positive intestinal parasite result were communicated with the stake holders and treated in the study Health Centre.

#### Data collection and analysis

An interview based structured questionnaire was used to collect socio-demographic and other data. The recent CD4 cell count of patients was obtained from each study participant ART fellow-up record in the Health Center. Training was given for data collectors before data collection. There were also continues monitoring throughout the study period. Data quality was checked and were entered to Microsoft Excel and exported to SPSS version 20 software and analysed. Binary logistic regression was done to investigate the relationship between the dependent and explanatory variables. P < 0.05 was considered statistically significant.

#### Stool collection and examination

Stool specimen collected from each patient was examined by direct wet mount method using normal saline (0.85% NaCl solution) at Kombolcha Health Center Laboratory. Lugol’s iodine was used to detect the cyst of intestinal protozoan parasites. The remaining sample was preserved with 10% formalin and examined by formol-ether concentration technique and modified Zeihl–Neelsen method.

#### Formol-ether concentration technique and Modified Ziehl–Neelsen method procedures

Formol-ether concentration technique was performed from each stool specimens collected from the study participants. Using a stick, an estimated 1 g (pea-size) of representative faeces was emulsified in about 4 ml of 10% formol water contained in a screw-cap tube. Then further 4 ml of 10% v/v formol water was added and mixed well by shaking. The emulsified faeces were sieved and 4 ml of diethyl ether was added. The tube was mixed for 1 min and immediately centrifuged at 3000 revolution per minute (rpm) for 1 min.

After centrifugation, the sediment in the bottom of the tube was transferred to a slide and covered with a cover glass. Then the preparation was examined microscopically using the 10× and 40× objective lenses.

Smear from the remaining sediment was stained using Modified Ziehl–Neelsen method. The smear was stained with Carbol fuchsin for 15 min after air dried and fixed with methanol for 2–3 min. The stain was decolorized with 1% acid alcohol for 15 s and counterstained with methylene blue for 30 s.

### Results

A total of 223 HIV sero-positive individuals who are on ART in Kombolcha Health Centre were examined for intestinal parasites. Of the total study participants 153 (68.6%) were females, 145 (65.0%) were between 30 and 49 age group, 205 (91.9%) were urban resident, 116 (52.0) were married and the educational status of majority of them 108 (48.4%) was elementary level (Table 1).

**Table 1:** Socio-demographic characteristics of HIV sero-positive patients who are on ART in Kombolcha Health Center ART clinic from June 2016 to August 2016

Variables	Category	Frequency	Percentage (%)
Sex	Female	153	68.6
	Male	70	31.4
Age (years)	10–29	46	20.6
	30–49	145	65.0
	≥ 50	32	14.4
Marital status	Widowed	40	17.9
	Unmarried	16	7.2
	Divorced	51	22.9
	Married	116	52.0
Occupation	House wife	39	17.5
	Governmental	42	18.8
	Daily labour	78	35.0
	Merchant	53	23.8
	Farmer	11	4.9
Residence	Rural	18	8.1
	Urban	205	91.9
Educational status	Illiterate	79	35.4
	Elementary	108	48.4
	High school and above	36	16.1

With regard to the clinical data of the study participants, majority of them had previous treatment for intestinal parasites. Eighteen of the 31 intestinal parasite positive HIV patients had previous treatment. Twenty five of the 31 intestinal parasite infected study participants had been on ART for more than 3 years. The CD4 counts of majority of the intestinal parasite infected and negative individuals were between 500 and 750 cells/μl (Additional file 1: Table S1).

The overall prevalence of intestinal parasites was 31/223 (13.9%). Nine different intestinal parasites species detected in this study were *Giardia lamblia*, *Entameoba histolytica*, Cryptosporidium species, *Isospora belli*, *Ascaris lumbricoides*, *Enterobius vermicularis*, *Hymenolepis nana*, Taenia spp. and *Strongyloides stercoralis*. The dominant intestinal parasite species was *E. histolytica*, which accounts 7.2% (16/223); followed by *G. lamblia* 3/223 (1.4%) and Cryptosporidium species 3/223 (1.4%) (Table 2).

**Table 2:** Parasites detected in HIV/AIDS patients who are on ART in Kombolcha Health Center from June 2016 to August 2016

Parasites detected	Number (%)
*Entameoba histolytica*	16 (7.2)
*Giardia lamblia*	3 (1.4)
Cryptosporidium spp.	3 (1.4)
*Isospora belli*	1 (0.5)
*Enterobius vermicularis*	1 (0.5)
*Ascaris lumbricoides*	3 (1.4)
*Hymenolepis nana*	1 (0.5)
Taenia spp.	2 (0.9)
*Strongyloides stercoralis*	1 (0.5)
Total	31 (13.9)

#### Risk factors for intestinal parasites in HIV/AIDS patients

Majority of study participants had the habit of washing their hand before meal and after toilet 215 (96.4%) and most of them didn’t use water treating chemicals 73 (32.7%). Most of the study participants 126 (56.5%) had private toilet. Binary logistic regression showed statistically significant association between rarely using water treating chemicals and intestinal parasitic infection in HIV patients (Table 3).

**Table 3:** Risk factors for intestinal parasitic infections among ART attendant HIV patients at ART clinic of Kombolcha Health Center from June 2016 to August 2016

Characteristics	Category	Total number	Intestinal parasitic infection		P-value	OR (95% CI)
			Positive [N (%)]	Negative [N (%)]		
Hand washing habit	Always	215 (96.4)	31 (13.9)	184 (82.5)		1
	Sometimes	8 (3.6)	0 (0.0)	8 (3.6)	0.90	
Utilization of water treating chemical	Always	39 (17.5)	7 (3.1)	32 (14.3)	0.18	0.5 (0.14–1.5)
	Sometimes	56 (25.1)	5 (2.2)	51 (22.9)	0.66	1.3 (0.4–4.5)
	Rarely	55 (24.7)	12 (5.4)	43 (19.3)	0.03	0.4 (0.1–1.1)
	Never	73 (32.7)	7 (3.1)	66 (29.6)		1
Type of toilet	Private	126 (56.5)	21 (9.4)	105 (47.1)	0.99	1.0 (0.1–10.1)
	Public	90 (40.4)	9 (4.0)	81 (36.3)	0.59	1.9 (0.2–19.7)
	No toilet	7 (3.1)	1 (0.4)	6 (2.7)		1
Raw meat eating habit	Always	0 (0.0)	0 (0.0)	0 (0.0)	0.12	0.2 (0.0–1.6)
	Sometimes	6 (2.67)	2 (0.9)	4 (1.8)	0.39	0.6 (0.2–1.8)
	Rarely	24 (10.8)	5 (2.2)	19 (8.5)	0.45	0.7 (0.3–2.0)
	Never	193 (86.6)	24 (10.8)	169 (75.8)		1
Water source	Piped water	216 (96.9)	30 (13.5)	186 (83.4)		1
	Protected well	4 (1.8)	1 (0.4)	3 (1.3)	0.92	0.9 (0.1–10.3)
	Unprotected well	3 (1.5)	0 (0.0)	3 (1.3)	0.99	1.0 (0.2–12.1)
Utilization of raw vegetables and fruits	Frequently	30 (13.5)	4 (1.8)	26 (11.7)		1
	Sometimes	98 (44.0)	15 (6.7)	83 (37.2)	0.59	0.7 (0.2–2.5)
	Rarely	44 (19.7)	5 (2.2)	39 (17.5)	0.58	0.7 (0.1–3.0)
	Never	51 (22.9)	7 (3.1)	44 (19.7)	0.68	0.7 (0.2–3.1)

### Discussion

Intestinal parasites are endemic in many regions of the world where HIV/AIDS is also prevalent [2]. This study reports the prevalence of intestinal parasites among HIV sero-positives visiting Kombolcha Health Center ART clinic for follow up of their CD4 status and ART treatment. In this study, majority of the participants (68.6%) were females which were in agreement with a report from Sokoto, Nigeria [9]. The biological differences between men and women may account for this, as men can infect women more easily than women can infect men. In addition, being the receptive part in heterosexual intercourse is believed to contribute to increased prevalence of infection in women [9]. During sex semen enters the vagina, where it can stay for several hours, increasing the risk of infection. The virus enters the bloodstream via tiny abrasions that form in the sensitive lining of the vagina during intercourse. Another contributing factor might be transactional sex, due to their unequal power in the exchange. Majority of the patients in this study were married (52.0%). This could be related to the cultural predisposition to early marriage in the predominantly Muslim population in the study area.

In the present study the prevalence of intestinal parasites among HIV/AIDS patients was 13.9%. This prevalence was lower than the cross-sectional study conducted at Hawassa Teaching and Referral Hospital and ART clinic of Arbaminch hospital [10, 11]. The prevalence was higher than the prevalence reported in Jos (9.5%) [12]. This might be due to the difference in study area, occupation and awareness. In the above mentioned studies most of the study participants were involved in farming occupation which might expose them to contaminated soil.

Other possible reasons for difference in prevalence might be due to variation in study period, scheduled deworming program to people taking ART and exposing life styles. In addition, study participants might have better awareness and practice for the prevention and control of intestinal parasites. For instance, in the present study majority of the study participants had the habit of using water treating chemicals.

On the other hand, the prevalence of intestinal parasites in the present study was the same with a report from Nekemte [13]. In our study, the most prevalent parasite detected was *Entameoba histolytica* which was consistent with previous study done in Abuja [14]. In contrast to our finding *G. lamblia* was the dominant parasite detected in the ART clinic of Arbaminch Hospital [10].

### Conclusions and recommendations

Although, the prevalence of intestinal parasites in the study area is lower than reports from other areas, periodic laboratory stool specimen examination, awareness creations about the prevention and control of intestinal parasites and prompt treatment are necessary. Simultaneously, utilization of water treating chemicals by HIV patients should be promoted.

## Limitations

Due to lack of resources in this research special technique for the detection of *Entrobious vermicularis*, *Strongyloides stercoralis* and *Schistosoma mansoni* was not performed which would help to increase the detection of this helminthes.

## Additional file

**Additional file 1: Table S1.** Clinical data of ART attendant HIV patients who attend at ART clinic of Kombolcha Health Center from June 2016 to August 2016.

## Abbreviations

AIDS: acquired immunodeficiency syndrome
ART: anti-retroviral therapy
CD4: cluster of differentiation on T_4_ lymphocyte cells
HIV: human immunodeficiency virus
