# Supplementary material for: Intestinal parasitosis among HIV/AIDS patients who are on anti-retroviral therapy in Kombolcha, North Central, Ethiopia: a cross-sectional study
Source: BMC Res Notes. 2018 Aug 25;11:613. doi: 10.1186/s13104-018-3726-6 (PMC6109261; doi:10.1186/s13104-018-3726-6)
Supplement: Supplementary file 1 — Additional file 1: Table S1. Clinical data of ART attendant HIV patients who attend at ART clinic of Kombolcha Health Center from June 2016 to August 2016. [file 13104_2018_3726_MOESM1_ESM.docx]

**Additional files**

**Additional file 1**

**Table S1:** Clinical data of ART attendant HIV patients who attend at ART clinic of Kombolcha Health Center from June 2016 to August 2016

| Characteristics | Category | Total N (%) | Intestinal Parasitic infection | |
| --- | --- | --- | --- | --- |
|  |  |  | Positive (N(%)) | Negative (N (%)) |
| Treatment for IP ever before | Yes | 116 (52.02) | 18 (15.5%) | 98 (84.5) |
|  | No | 107 (47.98) | 13 (12.1) | 94 (87.9) |
| ART started | <1 year | 20 (8.97) | 4 (20) | 16 (80.0) |
|  | 1. years | 23 (10.31) | 2 (8.7) | 21 (91.3) |
|  | 3 years | 9 (4.04) | 0 (0.0) | 9 (100.0) |
|  | > 3 years | 171 (76.68) | 25 (14.6) | 146 (85.4) |
| CD4 count | < 200 | 11 (4.93) | 2 (18.2) | 9 (81.8) |
|  | 200-499 | 80 (35.87) | 8 (10.0) | 72 (90.0) |
|  | 500-750 | 90 (40.36) | 15 (16.7) | 75 (83.3) |
|  | >750 | 42 (18.83) | 6 (14.3) | 36 (85.7) |
